# Description of A/C gene mutation related dilated cardiomyopathy with gadolinium- enhanced magnetic resonance imaging

**DOI:** 10.1186/1532-429X-13-S1-M5

**Published:** 2011-02-02

**Authors:** Miia Holmström, Sari Kivistö, Tiina Heliö, Raija Jurkko, Maija Kaartinen, Margareta Antila, Eeva Reissell, Johanna Kuusisto, Satu Kärkkäinen, Keijo Peuhkurinen, Juha Risteli, Juha Koikkalainen, Jyrki Lötjönen, Kirsi Lauerma

**Affiliations:** 1 Helsinki University Central Hospital, Helsinki, Finland; 2 Nordic Healthcare group, Helsinki, Finland; 3 Kuopio University Hospital, Kuopio, Finland; 4 University of Oulu, Oulu, Finland; 5 Institutes of and Clinical Medicine and Diagnostics, University of Oulu, Oulu, Finland; 6 VTT Technical Research Centre of Finland, Tampere, Finland

## Introduction

Dilated cardiomyopathy (DCM) is a major cause of heart failure and sudden cardiac death. About one third of DCM is familial. Several DCM disease genes have been identified, many of them limited to only single individuals or families. A/C gene (LMNA) is sofar the most significant disease gene of DCM. Cardiac magnetic resonance imaging (MRI) plays an important role in characterization and risk stratification of patients with DCM. About one third of DCM patients have demonstrated mid-myocardial linear enhancement on DE-MRI in a non-coronary distribution, due to fibrosis.

## Purpose

The purpose of this study was to identify myocardial delayed enhancement (DE) pattern, regional wall motion abnormalities, ventricular volumes and function with cardiac MRI in asymptomatic or mildly symptomatic carriers of LMNA mutations causing DCM. Secondly, we investigated the possible association between localization of myocardial fibrosis in MRI and conduction abnormalities documented with electrocardiography.

## Methods

Seventeen asymptomatic or mildly symptomatic LMNA mutation carriers and 14 healthy controls underwent cardiac MRI. DE-MRI was performed to evaluate myocardial fibrosis. The location, pattern and extent of DE in the left ventricular myocardium were visually estimated. Cine MRI was performed to study regional wall motion and global function of ventricles.

## Results

**Figure 1:**
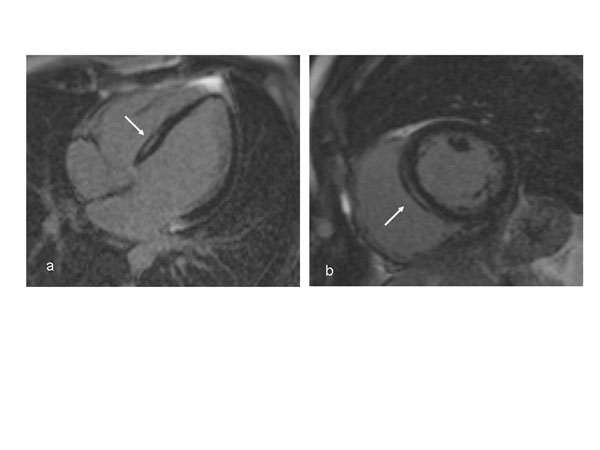
Delayed enhanced cardiac MRI of a 32- year old male with A/C lamin mutation DCM. Four-chamber (a) and short axis (b) views of the heart show typical mid-myocardial and linear enhancement of the basal septum.

Out of 17 patients, 15 exhibited myocardial fibrosis (88%). Among the total of 289 myocardial segments, DE was observed in 47 (16%). In all patients DE occurred in the basal or mid-ventricular septal wall (Figure 1). Fibrosis caused segmental wall motion abnormalities and correlation was strong when compared to the degree of DE (p<0,001) (Table 1). Myocardial DE associated with conduction abnormalities. Eleven patients with conduction abnormalities and two with atrial fibrillation had enhancement in the basal and mid-ventricular septum. Significant LV ventricular dilatation and decrease in systolic function in both ventricles was found compared to controls (Table 2).

**Table 1:** Myocardial delayed enhancement (DE) and movement abnormalities in the left ventricle

Percentage of DE in segments	Normal movement (%)	Abnormal movement (%)	Total
No enhancement	240 (99)	2 (1)	242

0-25%	12 (75)	4 (25)	16
26-50%	10 (50)	10 (50)	20
51-75%	0 (0)	6 (100)	6
76-100%	0 (0)	5 (100)	5

Total	262	27	289

**Table 2:** The Left ventricular volumes and function parameters evaluated with MRI.

	Males n=8	Females n=8	Controls n=14 (both females and males)
BSA m^2^	1.99 ± 0.20	1.80 ± 0.25	1.80 ± 0.2
LV EDV ml/m^2^	109.83 ± 8.56	85.81 ± 11.54	80 ± 20
LV ESV ml/m^2^	52.40 ± 16.32	34.15 ± 4.10	24 ± 7
SV ml/m^2^	57.44 ± 14.97	51.65 ± 9.95	56 ± 13
EF%	52 ± 13	60 ± 0.05	70 ± 3

## Conclusions

In the early stage of DCM caused by a lamin A/C gene mutation cardiac conduction abnormalities and mildly depressed LV function are common but also other cardiac diseases, like sarcoidosis, may produce a similar phenotype. Cardiac MRI is an accurate tool to determine typical cardiac involvement in the primal state of LMNA cardiomyopathy and may help to initiate early treatment.

